# Psychoinflammation: a conceptual framework for peripheral inflammatory phenotypes in mental health

**DOI:** 10.3389/fpsyt.2026.1775578

**Published:** 2026-03-11

**Authors:** Aslı Kazgan Kılıçaslan

**Affiliations:** Department of Psychiatry, Buca Seyfi Demirsoy Training and Research Hospital, İzmir, Türkiye

**Keywords:** etiology, immune system, psychoinflammation, psychoneuroimmunology, inflammation

## Introduction

Broad umbrella concepts used to explain the relationship between psychological experiences and inflammatory markers carry a risk of conceptual clutter (1). Terms such as psychoneuroimmunology, immunopsychiatry, psychosomatics, and neuroinflammation share a conceptual ground, with each emphasizing a different level or direction; however, they contain uncertainties regarding the causal starting point. In this study, the term “psychoinflammation” is introduced as a more mechanistic taxonomy to explain phenotypes in which mental processes initiate a clearly measurable inflammatory response in the immune system.

Psychoinflammation refers to a peripheral-dominant inflammatory biotype within the inflammatory continuum, where etiological priority is attributed to a psychogenic trigger. This biotype can be more distinctly demonstrated clinically in cases in which central inflammatory involvement is not dominant or is not demonstrable.

## Definition and scope of the psychoinflammation concept

“Psycho” denotes psychological experience, conscious content, and behavioral phenotypes. Operationally, it represents phenomena measured through validated scales, such as stress, trauma, or an individual’s subjective experience. Stress here refers to “perceived stress”, where the individual interprets events as threatening or unmanageable rather than objective stressors. Although the subjective nature of psychological processes may appear to be a limitation in terms of measurement, these phenomena can be measured using valid and standardized tools. Instruments used in the measurement of psychological triggers, such as the Perceived Stress Scale and the Childhood Trauma Questionnaire, convert subjective experience into quantitative data (2, 3). On the other hand, the objective and measurable nature of inflammation allows for the scientific validation of the etiological link. Subjective experiences are significantly associated with objective physiological criteria such as the allostatic load index, diurnal cortisol rhythm, and heart rate variability (4, 5). In the validation study of the Perceived Stress Questionnaire, Fliege et al. (6) demonstrated significant correlations between subjective stress measurements and objective immune markers by showing that high stress scores were associated with immunological imbalances. In posttraumatic stress disorder, an algorithm based on serum cortisol and interleukin (IL)-6 levels was developed, and it was reported that this algorithm could distinguish patients with a 78% accuracy rate (7). It has been shown in COPD patients that high stress levels measured by the Perceived Stress Scale are distinctly associated with specific biological pathways such as platelet activation (urinary 11-dehydro-thromboxane B2) and oxidative stress (8-isoprostane) (8). Acute social stress protocols (social pressure/stress stimulus) have been shown to produce robust effects on many inflammatory markers such as IL-5, IL-22, IL-8, and IL-10 (9). All of this is evidence demonstrating that psychological stress can be associated not only with subjective scores but also with biological pathophysiological markers. Despite all this evidence, methodological challenges regarding the standardization of subjective psychological measurements persist. Therefore, as mentioned above, the concept of psychoinflammation requires a multidimensional assessment based on the exclusion of central nervous system findings, in addition to the objective presence of peripheral inflammation markers.

The critical point of this term is that it prioritizes the “psychological” trigger. In this respect, it offers a more mechanistic approach than the term “psychosomatic” and a clearer, distinct etiological starting point compared to the term “neuroinflammation”. Attributing causal primacy to the psychological trigger is supported by experimental evidence, such as the rapid increase observed in inflammatory markers following acute stress exposure and the decrease in these markers following psychological interventions. Simultaneously, “psycho” indicates the “level” at which the phenomenon is observed. The aim of the term is not to create a new nosological category but to define a specific etiological biotype within existing psychopathologies. In this sense, psychoinflammation defines cases that “demonstrate the effects of psychological processes through inflammation” and proposes a narrowed subset focused on the mechanism.

The existing evidence in the literature directs us to the criteria listed below. In clinical practice, psychoinflammation can be measured as follows. Furthermore, its empirical distinguishability and falsifiability are critical to the methodological strength of the concept.

1. Trigger: The presence of subjective psychological triggers or a history of a demonstrable intensity in the recent past; a significant level of psychological load validated by standardized scales. In the differential diagnosis of this biotype, the severity of inflammation is expected to correlate positively with psychological stress scores, while this relationship should be weakly correlated with metabolic parameters.
2. Biological evidence: Observation of high levels of peripheral inflammatory markers. In their research using a “transdiagnostic inflammatory subgroup” approach, Byrne et al. (10) identified an “inflamed” biotype determined through cluster analyses in which multiple inflammatory markers were evaluated together. Cluster analyses using these criteria may enable the differentiation of the psychoinflammation subtype from other inflammatory subtypes (e.g., inflammation related to autoimmunity or metabolic syndrome). For instance, high-sensitivity CRP > 3 mg/L is a practical criterion used in most studies to define a “high inflammation” subgroup (11). Psychoinflammation uses the requirement of a “psychogenic initiator” as a methodological filter to distinguish this inflammatory subgroup from others.
3. Central nervous system (CNS) dissociation: Incongruence between central inflammation and peripheral load, or its persistence in the secondary background. Heterogeneity of clinical symptoms and the dominance of peripheral biomarkers are frequently reported in the literature. Within current technological limitations, this dissociation can be demonstrated using neuroimaging [e.g., Translocator protein positron emission tomography (TSPO-PET)], as well as by the absence of neurological findings specific to central inflammation and the primary contribution of peripheral inflammatory markers to the clinical picture. This criterion represents the peripheral-dominant configuration in which the model is limited to the peripheral compartment, as peripheral signals in this configuration cannot yet be associated with demonstrable parenchymal activation (12).
4. Psychological processes as the primary source of inflammatory load: Clinical exclusion of dominant alternative explanations for inflammation, such as active infection, cancer, or autoimmune diseases, with these conditions remaining secondary in explaining the current peripheral inflammatory picture. Psychoinflammation must be falsifiable at this point. If inflammatory markers remain elevated despite the reduction or control of the psychological burden, and if evidence of neuroinflammation coinciding with peripheral inflammatory load can be demonstrated at this stage, this clinical picture should be considered outside the scope of psychoinflammation. In this regard, psychoinflammation necessitates a multistage exclusion process. The inflammatory subgroup should be identified through multivariate classification or clustering methods. Subsequently, it must be tested whether the correlation between psychological load and inflammation is significantly stronger than its association with other metabolic parameters (insulin resistance, BMI, etc.). Thus, it transforms into an empirically distinguishable concept.

These criteria represent an increasingly important approach to stratifying patient populations into more homogeneous subgroups. Indeed, the utilization of these criteria in determining inflammatory biotypes is steadily rising. Notably, a recent study using cluster analysis in patients with major depressive episodes identified three distinct immunological profiles and succeeded in distinguishing patients from healthy controls with an 83.8% accuracy rate using the Random Forest model. The cluster with the strongest inflammatory profile identified in this study (cluster 3) was characterized by high levels of tumor necrosis factor alpha (TNF-α), CX3CL1, IL-12p70, IL-17A, IL-23, and IL-33, indicating an active inflammatory process. These findings demonstrate that peripheral immune phenotyping is a powerful tool that can be used to differentiate homogeneous subgroups within mood disorders (13). Researchers can identify patients who belong to the psychoinflammation biotype using these criteria. In this way, the efficacy of anti-inflammatory treatments can be tested more precisely in patients who belong to this biotype compared with those who do not. In clinical practice, screening for these criteria in cases of treatment nonresponsiveness allows the explanation of treatment resistance through the “inflammatory barrier” hypothesis and promotes integrated approaches focusing on stress management.

Additionally, although psychoinflammation overlaps with low-grade inflammation (LGI) in some aspects, it is distinct from it. LGI refers to systemic, chronic, low-intensity, subclinical inflammation with many potential determinants, including lifestyle, aging, and stress. Psychoinflammation, however, aims to distinguish a peripheral-dominant biotype methodologically within this heterogeneous cluster, where the inflammatory response is linked to a psychogenic trigger with causal priority and metabolic or autoimmune explanations remain secondary. Its priority is not the severity of inflammation, but rather the initial direction and etiological precedence of the inflammatory process.

Psychoinflammation, however, aims to delineate methodologically a peripheral-dominant, potentially early-stage biotype within this heterogeneous cluster, in which the inflammatory response is hypothesized to be linked to a psychogenic trigger with causal precedence, while metabolic or autoimmune explanations are not considered primary. Its focus is not the severity of inflammation but rather the initial direction and proposed etiological ordering of the inflammatory process.

The scientific validity of psychoinflammation is based on measurable and reproducible biological processes, namely, inflammatory pathways. However, the first step of the process, the “psycho” component, involves neurobiological conversion mechanisms that are not yet clearly understood and require further research. The theoretical strength of the term lies in offering an etiological classification. This term is not a concept that fully explains “how” psychological experiences are transformed into neurobiological signals at the “initial moment”. The mechanistic refinement provided by the term focuses not on the details of the initial steps, but on confirming the inflammatory pathway as the primary pathogenic mechanism in subgroups with psychological etiology. The most significant evidence for the validity of the term is the dissociation of peripheral inflammation from central inflammation observed in psychopathologies. However, peripheral and central inflammation are not entirely independent processes (14), and this point must be addressed in a balanced manner in light of the extensive literature demonstrating bidirectional communication between the peripheral and central immune systems. At the same time, the concept of psychoinflammation should evaluate this dissociation within a continuum where the psychological trigger primarily produces effects at the peripheral level and central reflections are temporally delayed or exhibit individual differences. Peripheral signals do not always leave a measurable and/or consistent trace centrally. For example, high inflammation markers in the peripheral blood in depression and schizophrenia are not always consistent with inflammation in the CNS, and results are generally heterogeneous. In individuals with psychosis, no significant differences were detected in brain levels of translocator protein, a marker of microglial activation, even when grouped according to peripheral inflammatory clusters (15). Although serum IL-6 levels were found to be elevated in patients with anxious depression, structural CNS findings, such as gray matter volume changes, were observed only in specific regions (16).

This empirical dissociation appears consistent with the hypothesis that the “psycho” component may manifest as peripheral inflammation and that neuroinflammation may be a secondary and/or variable consequence. Psychoinflammation is much more suitable for classifying disease subgroups driven by a systemic inflammatory biotype, representing the biological cost of psychological processes, in cases lacking CNS findings. In this way, the efficacy of specific anti-inflammatory and immunomodulatory treatments can be tested with higher sensitivity compared to etiologically more heterogeneous groups. The most concrete counterpart of the concept in clinical practice is its potential to provide a prognostic prediction. This term can serve as a “warning sign” for clinicians. It can offer the clinician the opportunity to rationalize the cause of treatment nonresponsiveness as a tangible “inflammatory barrier”. This provides clinicians with an integrated approach focused on stress management.

## Etiological and mechanistic critiques regarding existing terminologies

The fundamental basis for the idea of psychoinflammation is the causality ambiguity in the holistic approach of the term psychoneuroimmunology, the ambiguity in the term psychosomatic, and the site restriction in the term neuroinflammation (17).

The term psychoneuroimmunology assigns equal weight to each factor in its triple composite structure; the primary mechanism is not clear and involves bidirectional interaction. Researchers must control for many potential confounding factors to clarify the theoretical causal structure, which can reduce the sensitivity of the primary relationship. This low sensitivity has been demonstrated in causal relationship research (1), and the methodological importance of determining a clear etiological starting point in psychoneuroimmunology research has been emphasized (1). The focus on an extra-CNS initiator in psychoinflammation is a step toward introducing the principle of “causal priority”. However, this term does not reject the existence of a bidirectional cyclic relationship; on the contrary, it focuses on clarifying the starting point of the cycle. It should be considered a mechanistic tool that provides etiological clarity and points to a specific biotype, particularly for cases where peripheral inflammation is inconsistent with CNS findings (15). In this respect, psychoinflammation is not a new paradigm disconnected from psychoneuroimmunology; rather, it attempts to conceptually clarify this network. Psychoinflammation refers to an etiologically defined peripheral-dominant biotype within the inflammatory continuum, particularly in the early stages where peripheral components predominate (1, 10). In doing so, it provides methodological precision to overcome the causal heterogeneity in psychoneuroinflammation (1, 17).

The concept of immunopsychiatry, as an alternative to the equally weighted structure of psychoneuroimmunology, offers a hierarchical model that positions dysfunctions in the immune system as the primary cause and psychiatric conditions as secondary consequences. As emphasized by Pariante (18), this approach focuses on the driving effect of peripheral immune mechanisms on behavior and mood. However, this approach fails to explain cases where psychological processes trigger the inflammatory response; in other words, it establishes the causal arrow in the exact opposite direction. One of the greatest paradoxes in the field of immunopsychiatry is that inflammatory markers in the peripheral circulation do not always reflect the CNS. This is one of the fundamental reasons for the heterogeneity observed in clinical research.

Neuroinflammation is indeed a process with complex, dynamic, and functional dimensions (19). In neuroinflammation, the target is clearly CNS processes, neuronal circuits, and brain functions. However, to intervene, we must observe the outcomes of the event, and we cannot answer the question of why the inflammation began. Since it does not provide direct information about the origin of the inflammation, it can be defined as an anatomically localized and advanced-stage consequence. Psychoinflammation comes into play precisely at this point. By focusing on the systemic and peripheral stage of the process initiated by a psychological trigger in the CNS, it offers a potential etiological explanation for how and why neuroinflammation emerges before structural damage occurs and in the absence of detectable structural changes.

As shown by Plavén-Sigray et al. (15), in patients with psychosis, the presence of peripheral inflammation does not always overlap with microglial activation in the CNS. The blood–brain barrier (BBB) is not merely a mechanical obstacle but is also a dynamic immune modulator that functions as a filter (20). Although peripheral cytokines such as IL-1β, IL-6, and TNF-α can transmit signals to the brain through circumventricular organs or the vagus nerve, they do not always directly infiltrate the brain parenchyma. This situation does not always result in chronic microglial activation or neuroanatomical damage within the brain parenchyma. Contrary to the view that systemic inflammation inevitably leads to neuroinflammation, recent evidence suggests that the BBB and the brain’s compensatory mechanisms function as a gatekeeper. Signals traveling from the periphery to the brain sometimes remain only at the level of neurotransmitter modulation. This constitutes communication but is not neuroinflammation, characterized by tissue damage or intense glial activation. In psychoinflammation, peripheral inflammation exists, but this stimulus threshold may not have yet reached the level to completely cross the BBB or chronically activate the microglia. It defines the critical interval where the systemic load has not yet exceeded the repair capacity of the brain or where the integrity of the BBB is maintained. At this point, psychoinflammation does not ignore the CNS; it refers to a systemic load that surrounds the CNS from the outside. Here, the CNS serves as a “functional bridge”; that is, it is not a structural impairment or a primary focus of inflammation. What is excluded is not the presence of neurobiological translation steps such as Hypothalamic–Pituitary–Adrenal (HPA) axis activation or autonomic nervous system activation; rather, it is the CNS-weighted outcome that defines neuroinflammation. Therefore, the exclusion of the neuro prefix does not reject or exclude existing neurobiological steps. It does not deny the dynamic nature of neuroinflammation. It prioritizes the non-CNS starting point to identify cases where the inflammatory load has not yet converted, or may not convert, into structural or chronic central neuroinflammation.

The psychosomatic approach is one of the first scientific frameworks describing the relationship between the body and the mind. However, for many years, this approach was unable to clearly describe biological mechanisms and has often remained mechanistically independent. While it indicates that a physical symptom originates from emotional factors, it does not emphasize molecular details (21). The traditional term psychosomatic has, to a certain extent, ignored biological mediators and proceeds from the mind to the body.

The highly inclusive biopsychosocial model is subject to criticisms of “lack of specific mechanistic directionality” (22). It is argued that it fails to provide a sufficient conceptual explanation for the causal interactions between biological, psychological, and social domains and instead offers a general framework suggesting that everything is interrelated (22). In practical application, it is argued that a causal hierarchy cannot be established between biological reductionism and social expansion, which deprives the clinician of a concrete intervention route. Roberts (23) has asserted that this situation pushes researchers to produce speculative interpretations and leads to conceptual instability for medical research. In this context, psychoinflammation adds a concrete and mechanistic route to the abstract nature of the biopsychosocial model at the level where everything is related. Psychoinflammation is a specific, testable etiological hypothesis with a definite biological (inflammatory) path situated within the framework of the biopsychosocial model. In this respect, the biopsychosocial model is a general map, and psychoinflammation is a specific route on this map.

On the other hand, LGI represents a chronic, low-level increase in cytokines in the absence of infection, serving as a common denominator in many chronic diseases. It reflects a general chronic systemic load where metabolic factors, aging, physical factors, and lifestyle factors form a heterogeneous ground (24, 25). Psychoinflammation, however, diverges from LGI at the point of etiological specificity by proposing that the primary trigger is directly psychogenic. In LGI, the distinction between central and peripheral inflammation does not exist and is not required (10, 24). Therefore, psychoinflammation is also methodologically more homogeneous than LGI; it positions inflammation in a predominantly peripheral character, and this distinction is its mechanistic identifier. Unlike LGI, the novelty of the term is etiological (psychogenic trigger) and methodological (biotype) rather than nosological. The term psychoinflammation is consistent with views suggesting that psychological stress affects neurobiological processes through inflammatory mechanisms (26). The relationship between stress and inflammation is on a continuum. Psychoinflammation is a specific pattern within this continuum; in this respect, rather than being a general stress response, it defines a subset that has developed into an inflammatory biotype.

In this framework, the concept of psychoinflammation diverges from each of the existing terms at specific points. These distinctions are summarized in **Table 1**.

**Table 1:** Comparison of psychoinflammation and other concepts.

Concept	Primary focus	Causal primacy and flow	Critical gaps and explanatory challenges
Psychoneuroimmunology	Psychoneuroimmune interaction network	Bidirectional–uncertain	High variability, confounding factors, and low causal sensitivity
Immunopsychiatry	Effect of the immune system on psychiatric symptoms	Bottom–up: inflammation → psychopathology	Excludes the psychological trigger, fails to explain cases where psychological processes trigger the inflammatory response
Neuroinflammation	Inflammation in the CNS (microglial activation and neurovascular processes, etc.)	Outcome-oriented, CNS-weighted biological status	Does not cover the peripheral onset and provides no information about the origin of inflammation
Psychosomatic approach	Somatic projection of mental processes on physical diseases	Uncertain/general, usually independent of mechanism	Lack of specific mechanistic explanation regarding biological mediators
Biopsychosocial model	Synthesis of biological, psychological, and social determinants	Holistic, nonhierarchical	“Lack of mechanistic directionality” and conceptual instability are open to speculative interpretations
Low-grade inflammation (LGI)	Chronic systemic load due to metabolic and physical causes	Independent of etiology/general (obesity, aging, physical inactivity, etc.)	Low etiological specificity/does not offer a specific starting point and does not provide information regarding the “cause”.
Psychoinflammation	Measurable inflammation initiated in the periphery by a psychological trigger	Top–down: psychological trigger → peripheral inflammation	Mechanisms at the initial moment of psycho–neuro conversion are not yet fully explained

CNS, central nervous system.

## Discussion

Empirical findings suggest that elevations in peripheral inflammatory markers do not consistently correspond to measurable indicators of central neuroinflammation (12).

This dissociation is supported by significant empirical data. Current comprehensive reviews emphasize that the neuroinflammation process requires increased BBB permeability as well as glial and autonomic activation. When these pathways are not activated, inflammation may remain in the periphery (27). The literature reports that the effect of peripheral inflammation on the CNS is regional and selective; therefore, if certain brain regions or pathways are not affected, clinically significant neuropsychiatric symptoms or evidence of structural neuroinflammation may not be observed (27). Medical conditions such as advanced age, obesity, hypertension, and diabetes create low-grade chronic inflammation and make the brain vulnerable; these are referred to as silent contributors. It is known that, in the absence of these factors, peripheral inflammation of the same severity may not lead to neuroinflammation (28). Additionally, it has been demonstrated that molecules such as serotonin in the periphery can alter peripheral immune cell migration without affecting endothelial cell adhesion molecules (29). On the other hand, the immune response triggered by psychological stress is signaling-oriented, involving DAMPs and neuroendocrine axes rather than being driven by tissue damage (30). Research by Ramani et al. (31) has shown that noncolonizing bacteria in the gut can suppress peripheral inflammation in disease models such as psoriasis and atopic dermatitis. The effects of gut-derived signals, primarily through the peripheral immune system, demonstrate peripheral inflammation that is independent of the CNS.

Contrary to the traditional view, the model proposed by Turkheimer et al. (32) argues that brain barriers (the BBB and the blood-CSF barrier), rather than microglia, play the primary role in the development of psychiatric symptoms. According to this model, in the early stages, when peripheral inflammation is dramatic, the homeostatic disruption occurring in the brain barriers is sufficient to trigger sickness behavior and depressive symptoms long before microglial activation. This situation indicates that cases in which neuroinflammation cannot be demonstrated within methodological and sensitivity limits are actually under an “immunological siege” of the brain, although this siege has not yet converted into parenchymal inflammation. Temporary reductions in barrier permeability or metabolite dysfunctions, such as those involving the kynurenine pathway, do not require microglial activation to damage neuronal circuits. This indirect relationship model provides a biological basis for why peripheral and central inflammation frequently appear desynchronized (32, 33). Peripheral inflammation triggered by psychological stress in psoriasis (mediated by S100A7, A8/A9) is well known. However, whether this peripheral fire leads to neuroinflammation (an increase in S100B) remains unanswered. Current data suggest that peripheral S100 proteins do not cross directly into the CNS and that, as proposed by Turkheimer et al. (32), the protective mechanisms of the BBB remain active in these patients.

Extensive studies conducted by Maccioni et al., (2024 and 2025) (34, 35) have proven that systemic inflammation actually reduces the entry of PET tracers into the brain. This finding directly explains the paradox in clinical practice. The more dramatic and evident the peripheral inflammation in a patient, the more the BBB exhibits closure or changes in transport dynamics (36). Turkheimer et al. (37) observed no correlation between central and peripheral immunity in TSPO-PET examinations in major depressive disorder. In another study, although CRP was high as a peripheral inflammation marker in PTSD patients, microglial activation decreased in brain PET imaging. The authors interpreted this finding as indicating that stress-related peripheral inflammation is associated with a lack of detectable brain microglial activation (38). In patients with new-onset schizophrenia, IL-6 levels in plasma and CSF samples were found to be significantly higher than in the control group; however, no significant difference was detected in the TSPO marker in brain PET scans of the same patients (39). Meta-analyses conducted in major depression cohorts reveal that, in many patients with high CRP levels, brain TSPO binding does not differ from that of the control group. In depression cases with a CRP level above 3 mg/L, no linear correlation has been found between the severity of brain parenchymal inflammation and the severity of systemic inflammation. This finding represents the scientific literature counterpart of the observation in clinical practice (12). Despite the elevation of serum IL-6 in anxious depression, gray matter changes remained limited to specific brain regions (16). In their meta-analysis, Baumeister et al. (40) demonstrated that childhood traumas are strongly associated with high CRP, IL-6, and TNF-α in adulthood. The researchers have shown that peripheral inflammation of psychogenic origin can follow a course independent of the central system. However, it should be noted that the absence of CNS findings, such as a lack of TSPO-PET signal, should not be interpreted as the absolute absence of CNS–immune interaction within current methodological limits; rather, it should be viewed as a configuration dominated by the peripheral compartment, where central immune signals remain below the detection threshold.

Another reason why peripheral inflammation does not manifest centrally is the processing of inflammatory signals within border regions before they reach the brain parenchyma. Recent studies have revealed that calvarial bone marrow and parameningeal spaces serve as intermediate hubs in the interaction between peripheral and central immunity. PET imaging studies have determined that TSPO expression in the calvarial bone marrow and dural sinuses (especially at the confluence of sinuses) in individuals with major depression correlates with both peripheral cytokines and central immune markers. However, interestingly, peripheral and central parenchymal markers are not directly related to each other. This situation suggests that the major component of inflammation is concentrated in areas just outside the brain rather than leaking into the brain parenchyma (41). In chronic social stress models, while intense accumulation of neutrophils is observed in the meninges, the same stress has been shown not to lead to immune cell infiltration into the brain parenchyma. Nevertheless, activity in this border region continues to profoundly affect brain function and mood through cytokine signaling (41, 42).

On the other hand, when psychological triggers are involved, dissociative symptoms and early life traumas play a key role in shaping the inflammatory response. Dissociation is known to increase CRP levels via the HPA axis. However, in dissociative individuals, the counterpart of CNS symptoms in the brain is generally not microglial activation but rather the functional desynchronization of neural circuits (43, 44). Could dissociation itself serve as a form of barrier modulation that restricts the entry of peripheral immune signals into the CNS as a defense mechanism of the brain? Although this is not yet known, the “high systemic–low central” inflammation profile observed in PTSD and dissociative subtype depression cases strengthens hypotheses in this direction (42, 45).

In clinical practice, when cases with prominent peripheral inflammation and uncertain neuroinflammation are encountered, this should be evaluated not as a false-negative result but as a specific stage or type of pathophysiology. Research shows that anti-inflammatory treatments are particularly effective in this group with prominent peripheral inflammation (41).

These serve as evidence that the effects of psychological triggers can create a biological cost manifested at the systemic level. Psychoinflammation was conceived to reduce the risk of etiological specificity being masked within complex cycles in existing concepts (17). In cases where the onset is psychological, peripheral inflammation is prominent, and central findings are inconsistent or unclear, psychoinflammation can provide a more precise explanatory framework than existing general terms. This will enable us to progress toward biologically defined patient biotypes and personalized medicine.

The most significant clinical contribution of the concept of psychoinflammation is the opportunity it offers to classify patients according to their biotypes rather than diagnostic labels. Traditional diagnostic methods, such as the DSM-5, group patients with biological differences into the same category. However, a patient with depression who has an inflamed biotype may benefit more from an anti-inflammatory intervention than from a standard antidepressant treatment. Research conducted by Donnelly et al. (2025) (46) has identified two fundamental latent components in depression: somatic-inflamed (high fatigue, sleep disturbance, high IL-6, and neutrophils) and anxious-noninflamed (high anxiety, low inflammatory markers). This distinction explains the current mixed results, as inflammation is the primary driver in one group of patients while it remains a secondary phenomenon or is relatively low in the other.

Psychoinflammation is not merely a new name; it is also a methodological call for research designs. Treating patients within this potential biotype as homogeneous groups can also help clarify findings and hypotheses.

Not all peripheral inflammatory responses progress toward central neuroinflammatory involvement. In some individuals, acute stress may cause significant peripheral inflammatory activation without measurable or clinically significant central effects. This configuration is conceptualized here as a peripheral-dominant biotype, rather than a temporal precursor to central inflammation. While some individuals may maintain this configuration as a chronic peripheral-dominant phenotype, in others, it may coexist with or transition into neuroinflammatory involvement. However, such a transition is not considered intrinsic or mandatory for the definition of the biotype.

## References

[1] Moriarity DP Mengelkoch S Slavich GM . Incorporating causal inference perspectives into psychoneuroimmunology: A simulation study highlighting concerns about controlling for adiposity in immunopsychiatry. Brain Behav Immun. (2023) 113:259–66. doi: 10.1016/j.bbi.2023.06.022, PMID: 37393056 PMC11225100

[2] Bernstein DP Stein JA Newcomb MD Walker E Pogge D Ahluvalia T . Development and validation of a brief screening version of the Childhood Trauma Questionnaire. Child Abuse Negl. (2003) 27:169–90. doi: 10.1016/s0145-2134(02)00541-0, PMID: 12615092

[3] Cohen S Kamarck T Mermelstein R . A global measure of perceived stress. J Health Soc Behav. (1983) 385–96. doi: 10.2307/2136404, PMID: 6668417

[4] Steptoe A Hamer M Chida Y . The effects of acute psychological stress on circulating inflammatory factors in humans: a review and meta-analysis. Brain Behav Immun. (2007) 21:901–12. doi: 10.1016/j.bbi.2007.03.011, PMID: 17475444

[5] McEwen BS . Protective and damaging effects of stress mediators. N Engl J Med. (1998) 338:171–9. doi: 10.1056/NEJM199801153380307, PMID: 9428819

[6] Fliege H Rose M Arck P Walter OB Kocalevent RD Weber C . The Perceived Stress Questionnaire (PSQ) reconsidered: validation and reference values from different clinical and healthy adult samples. Biopsychosoc Sci Med. (2005) 67:78–88. doi: 10.1097/01.psy.0000151491.80178.78, PMID: 15673628

[7] Zorkina Y Berdalin A Abramova O Reznik A Ushakova V Mukhin V . Serum cortisol and interleukin-6 as key biomarkers for a diagnostic algorithm of combat-related PTSD. Brain Sci. (2025) 15:1319. doi: 10.3390/brainsci15121319, PMID: 41440115 PMC12731115

[8] Offor O Eakin MN Woo H Belz D Williams M Raju S . Perceived stress is associated with health outcomes, platelet activation, and oxidative stress in COPD. Chronic Obstr Pulm Dis. (2025) 12:98. doi: 10.15326/jcopdf.2024.0561, PMID: 39933556 PMC12147820

[9] Hogenelst K Özsezen S Kleemann R Verschuren L Stuldreher I Bottenheft C . Seven robust and easy to obtain biomarkers to measure acute stress. Brain Behav Immun Health. (2024) 38:100789. doi: 10.1016/j.bbih.2024.100789, PMID: 38799794 PMC11126813

[10] Byrne JF Healy C Mongan D Susai SR Zammit S Föcking M . Transdiagnostic inflammatory subgroups among psychiatric disorders and their relevance to role functioning: a nested case-control study of the ALSPAC cohort. Transl Psychiatry. (2022) 12:377. doi: 10.1038/s41398-022-02142-2, PMID: 36085284 PMC9463145

[11] Pinzi M Fagiolini A Koukouna D Gualtieri G Rescalli MB Pierini C . Inflammatory and immune biomarkers in mood disorders: from mechanistic pathways to clinical translation. Cells. (2025) 14:1558. doi: 10.3390/cells14191558, PMID: 41090786 PMC12524191

[12] Schubert JJ Veronese M Fryer TD Manavaki R Kitzbichler MG Nettis MA . A modest increase in 11C-PK11195-PET TSPO binding in depression is not associated with serum C-reactive protein or body mass index. Biol. Psychiatry Cogn. Neurosci. Neuroimaging. (2020) 6:716–724. doi: 10.1016/j.bpsc.2020.12.017, PMID: 33515765 PMC8264953

[13] Daray FM Grendas LN Arena Á.R Tifner V Alvarez Casiani R Olaviaga A . Decoding the inflammatory signature of the major depressive episode: insights from peripheral immunophenotyping in active and remitted condition, a case–control study. Transl Psychiatry. (2024) 14:254. doi: 10.1038/s41398-024-02902-2, PMID: 38866753 PMC11169351

[14] Hosang L Flügel A Odoardi F . Body–brain axis: orchestrating immune responses. Cell Res. (2024) 34:757–8. doi: 10.1038/s41422-024-01004-4, PMID: 39014217 PMC11527975

[15] Plavén-Sigray P Matheson GJ Collste K Ashok AH Coughlin JM Howes OD . Positron emission tomography studies of the glial cell marker translocator protein in patients with psychosis: A meta-analysis using individual participant data. Biol Psychiatry. (2018) 84:433–42. doi: 10.1016/j.biopsych.2018.02.1171, PMID: 29653835 PMC7893597

[16] Vai B Palladini M Lorenzi C Zanardi R Poletti S Aggio V . Interleukin 6 associates with reduced grey matter volume and resting-state connectivity in the anterior cingulate cortex in bipolar patients. Brain Behav Immun Health. (2022) 26:100522. doi: 10.1016/j.bbih.2022.100522, PMID: 36187407 PMC9523275

[17] Androulakis IP . From cells to society: untangling the web of stress, inflammation, and social determinants of health. Front Sci. (2024) 2:1358784. doi: 10.3389/fsci.2024.1358784, PMID: 41789077

[18] Pariante CM . Neuroscience, mental health and the immune system: overcoming the brain-mind-body trichotomy. Epidemiol Psychiatr Sci. (2016) 25:101–5. doi: 10.1017/S204579601500089X, PMID: 26503420 PMC6998594

[19] Paolicelli RC Sierra A Stevens B Tremblay ME Aguzzi A Ajami B . Microglia states and nomenclature: A field at its crossroads. Neuron. (2022) 110:3458–83. doi: 10.1016/j.neuron.2022.10.020, PMID: 36327895 PMC9999291

[20] Daneman R Prat A . The blood–brain barrier. Cold Spring Harb Perspect Biol. (2015) 7:a020412. doi: 10.1101/cshperspect.a02041, PMID: 25561720 PMC4292164

[21] Kiecolt-Glaser JK McGuire L Robles TF Glaser R . Emotions, morbidity, and mortality: new perspectives from psychoneuroimmunology. Annu. Rev. Psychol. (2022) 53:83–107. doi: 10.1146/annurev.psych.53.100901.135217, PMID: 11752480

[22] Bolton D Gillett G . The biopsychosocial model 40 years on. In: The Biopsychosocial Model of Health and Disease: New Philosophical and Scientific Developments. Springer International Publishing, Cham (2019). p. 1–43. doi: 10.1007/978-3-030-11899-0_1 31886965

[23] Roberts A . The biopsychosocial model: Its use and abuse. Med Health Care Philos. (2023) 26:367–84. doi: 10.1007/s11019-023-10150-2, PMID: 37067677 PMC10107555

[24] Pérez-Castillo IM Rueda R Bouzamondo H Aparicio-Pascual D Valiño-Marques A López-Chicharro J . Does lifelong exercise counteract low-grade inflammation associated with aging? A systematic review and meta-analysis. Sports Med. (2025) 55:675–96. doi: 10.1007/s40279-024-02152-8, PMID: 39792347 PMC11985631

[25] Calder PC . Dietary factors and low-grade inflammation in relation to overweight and obesity revisted. Br J Nutr. (2022) 127:1455–7. doi: 10.1017/S0007114522000782, PMID: 35260214 PMC9044216

[26] Kocamer Şahin Ş Aslan E . Inflammation as a neurobiological mechanism of cognitive impairment in psychological stress. J Integr Neurosci. (2024) 23:101. doi: 10.31083/j.jin2305101, PMID: 38812387

[27] Sun Y Koyama Y Shimada S . Inflammation from peripheral organs to the brain: how does systemic inflammation cause neuroinflammation? Front Aging Neurosci. (2022) 14:903455. doi: 10.3389/fnagi.2022.903455, PMID: 35783147 PMC9244793

[28] Sandu RE Buga AM Uzoni A Petcu EB Popa-Wagner A . Neuroinflammation and comorbidities are frequently ignored factors in CNS pathology. Neural Regen Res. (2015) 10:1349–55. doi: 10.4103/1673-5374.165208, PMID: 26604877 PMC4625482

[29] Mauler M Schanze N Krauel K Schoenichen C Glatzki F Poeschl S . Peripheral serotonin lacks effects on endothelial adhesion molecule expression in acute inflammation. J Thromb Haemost. (2022) 20:222–9. doi: 10.1111/jth.15541, PMID: 34592035

[30] Ye X Ho YS Chang RC . Re-examination of inflammation in major depressive disorder: bridging systemic and neuroinflammatory insights. Biomolecules. (2025) 15:1556. doi: 10.3390/biom15111556, PMID: 41301474 PMC12650186

[31] Ramani K Cormack T Cartwright ANR Alami A Parameswaran P Abdou M . Regulation of peripheral inflammation by a non-viable, non-colonizing strain of commensal bacteria. Front Immunol. (2022) 13:768076. doi: 10.3389/fimmu.2022.768076, PMID: 35185874 PMC8847375

[32] Turkheimer FE Veronese M Mondelli V Cash D Pariante CM . Sickness behaviour and depression: An updated model of peripheral-central immunity interactions. Brain Behav Immun. (2023) 111:202–10. doi: 10.1016/j.bbi.2023.03.031, PMID: 37076054

[33] Han KM Ham BJ . How inflammation affects the brain in depression: A review of functional and structural MRI studies. J Clin Neurol. (2021) 17:503–15. doi: 10.3988/jcn.2021.17.4.503, PMID: 34595858 PMC8490908

[34] Maccioni L Michelle CM Brusaferri L Silvestri E Bertoldo A Schubert JJ . A blood-free modeling approach for the quantification of the blood-to-brain tracer exchange in TSPO PET imaging. Front Neurosci. (2024) 18:1395769. doi: 10.3389/fnins.2024.1395769, PMID: 39104610 PMC11299498

[35] Maccioni L Brusaferri L Barzon L Schubert JJ Nettis MA Cousins O . A novel blood-free analytical framework for the quantification of neuroinflammatory load from TSPO PET imaging. J Cereb Blood Flow Metab. (2025) 45:2283–300. doi: 10.1177/0271678X251361261, PMID: 40744907 PMC12316680

[36] Barzon L Maccioni L Moretto M Giacomel A Schubert JJ Cousins O . Peripheral inflammation is associated with reduced influx of TSPO PET tracers into the brain: insights from a non-invasive mapping methodology. (2025) doi: 10.21203/rs.3.rs-6648321/v2 42034206

[37] Turkheimer FE Althubaity N Schubert J Nettis MA Cousins O Dima D . Increased serum peripheral C-reactive protein is associated with reduced brain barriers permeability of TSPO radioligands in healthy volunteers and depressed patients: implications for inflammation and depression. Brain Behav Immun. (2021) 91:487–97. doi: 10.1016/j.bbi.2020.10.025, PMID: 33160089

[38] Bhatt S Hillmer AT Girgenti MJ Rusowicz A Kapinos M Nabulsi N . PTSD is associated with neuroimmune suppression: evidence from PET imaging and postmortem transcriptomic studies. Nat Commun. (2020) 11:2360. doi: 10.1038/s41467-020-15930-5, PMID: 32398677 PMC7217830

[39] Coughlin JM Wang Y Ambinder EB Ward RE Minn I Vranesic M . *In vivo* markers of inflammatory response in recent-onset schizophrenia: a combined study using [11C] DPA-713 PET and analysis of CSF and plasma. Transl Psychiatry. (2016) 6:e777–7. doi: 10.1038/tp.2016.40, PMID: 27070405 PMC4872398

[40] Baumeister D Akhtar R Ciufolini S Pariante CM Mondelli V . Childhood trauma and adulthood inflammation: a meta-analysis of peripheral C-reactive protein, interleukin-6 and tumour necrosis factor-α. Mol Psychiatry. (2016) 21:642–9. doi: 10.1038/mp.2015.67, PMID: 26033244 PMC4564950

[41] Eiff B Bullmore ET Clatworthy MR Fryer TD Pariante CM Mondelli V Schubert JJ . Extra-axial inflammatory signal and its relationship to peripheral and central immunity in depression. Brain. (2025) 148:635–646. doi: 10.1093/brain/awae343, PMID: 39657983 PMC11788198

[42] Calcia MA Bonsall DR Bloomfield PS Selvaraj S Barichello T Howes OD . Stress and neuroinflammation: a systematic review of the effects of stress on microglia and the implications for mental illness. Psychopharmacol (Berl). (2016) 233:1637–50. doi: 10.1007/s00213-016-4218-9, PMID: 26847047 PMC4828495

[43] Powers A Dixon HD Conneely K Gluck R Munoz A Rochat C . The differential effects of PTSD, MDD, and dissociation on CRP in trauma-exposed womenThe differential effects of PTSD, MDD, and dissociation on CRP in trauma-exposed women. Compr. Psychiatry. (2019) 93:33–40. doi: 10.1016/j.comppsych.2019.06.007, PMID: 31306866 PMC6689425

[44] Lanius RA Brand B Vermetten E Frewen PA Spiegel D . The dissociative subtype of posttraumatic stress disorder: rationale, clinical and neurobiological evidence, and implications. Depress. Anxiety. (2012) 29:701–8. doi: 10.1002/da.21889, PMID: 22431063

[45] Quinones MM Gallegos AM Lin FV Heffner K . Dysregulation of inflammation, neurobiology, and cognitive function in PTSD: an integrative review. Cogn. Affect. Behav. Neurosci. (2020) 20:455–480. doi: 10.3758/s13415-020-00782-9, PMID: 32170605 PMC7682894

[46] Donnelly NA Tsang RS Foley EM Fraser H Hanson AL Khandaker GM . Blood immuno-metabolic biomarker signatures of depression and affective symptoms in young adults. Brain Behav. Immun. (2025) 128:673–684. doi: 10.1016/j.bbi.2025.05.011, PMID: 40374127 PMC7619282

